# Reproductive awareness and recognition of unintended pregnancy: young women, key informants and health care providers perspectives in South Africa

**DOI:** 10.1186/s12978-021-01262-0

**Published:** 2021-10-26

**Authors:** Oluwaseyi Dolapo Somefun, Jane Harries, Deborah Constant

**Affiliations:** Women’s Health Research Unit, University of Cape Town, Anzio Road, 7925 Observatory, Cape Town, South Africa

**Keywords:** Pregnancy recognition, Unintended pregnancy, Reproductive awareness, Abortion care-seeking, South Africa

## Abstract

**Background:**

South Africa has a liberal abortion law, yet denial of care is not uncommon, usually due to a woman being beyond the legal gestational age limit for abortion care at that facility. For women successfully obtaining care, time from last menstrual period to confirmation of pregnancy is significantly longer among those having an abortion later in the second trimester compared to earlier gestations. This study explores women’s experiences with recognition and confirmation of unintended pregnancy, their understanding of fertile periods within the menstrual cycle as well as healthcare providers’ and policy makers’ ideas for public sector strategies to facilitate prompt confirmation of pregnancy.

**Methods:**

We recruited participants from July through September 2017, at an urban non-governmental organization (NGO) sexual and reproductive health (SRH) facility and two public sector hospitals, all providing abortion care into the second trimester. We conducted in-depth interviews and group discussions with 40 women to elicit information regarding pregnancy recognition and confirmation as well as fertility awareness. In addition, 5 providers at these same facilities and 2 provincial policy makers were interviewed. Data were analysed using thematic analysis.

**Results:**

Uncertainties regarding pregnancy signs and symptoms greatly impacted on recognition of pregnancy status. Women often mentioned that others, including family, friends, partners or colleagues noticed pregnancy signs and prompted them to take action. Several women were unaware of the fertility window and earliest timing for accurate pregnancy testing. Health care providers and policy makers called for strategies to raise awareness regarding risk and signs of pregnancy and for pregnancy tests to be made more readily accessible.

**Conclusion:**

Early recognition of unintended pregnancy in this setting is frustrated by poor understanding and awareness of fertility and pregnancy signs and symptoms, compounded by a distrust of commercially available pregnancy tests. Improving community awareness around risk and early signs of pregnancy and having free tests readily available may help women confirm their pregnancy status promptly.

## Plain language summary

South Africa has one of the world’s most progressive legal frameworks for abortion, yet it’s not uncommon for women to struggle to access safe abortion services. A key reason for this is late recognition of an unplanned pregnancy. This study explored the lived experiences leading up to pregnancy confirmation among women securing abortion care beyond 9 weeks gestational age, the legal limit for home use of medication for abortion. It fills a gap by also including providers’ and policy makers’ perspectives on ways to strengthen women’s prompt recognition of pregnancy. Using group discussions and in-depth interviews we elicited information from 40 women and 7 providers and policy makers in two health subdistricts in the Western Cape Province, South Africa. Our findings highlight the process of pregnancy recognition and confirmation and women’s knowledge of fertility, the menstrual cycle and when to test for pregnancy. Our results suggest that factors influencing women’s recognition of pregnancy are at the individual and at community level. Health care providers and policy makers suggested the use of community-based services to raise awareness around early pregnancy testing, and to expand easy access to self-testing outside the formal clinic setting as a mechanism to overcome clinic-based barriers.

## Background

Despite abortion being legal in South Africa, many women are still denied care when seeking abortion services. This has been largely attributed to being too far advanced in pregnancy at the time of presentation for care [1]. Studies have established multiple reasons associated with delays incurred during the care-seeking process which include structural barriers such as a scarcity of willing and trained providers [2], compounded by long distances to health facilities in rural areas [3]. At the individual level, irregular periods, underestimation of pregnancy likelihood, and delayed testing for pregnancy have been associated with obtaining abortion care later in pregnancy [4]. Furthermore, where abortion services are legal and available, stigma experienced within communities has resulted in reluctance to seek care close to home [5].

In their conceptual framework of trajectories to abortion, Coast et al. classify and link abortion-specific experiences to individual and national contexts to map factors influencing a woman’s journey to obtaining abortion care. These include time-dependent processes, such as the timing of pregnancy awareness [6]. Pregnancy awareness and confirmation are identified by Coast et al. as the initial steps in a woman’s journey to abortion care and as a domain in need of further exploration by researchers [6]. Earlier research had indicated that pregnancy discovery is a complex process, accompanied by ambiguity around pregnancy signs and symptoms, and varying risk perception of pregnancy [7]. These, together with the role of social networks in affirming pregnancy status all impacted significantly on the time to recognition of an unplanned pregnancy [7]. Interventions endorsed by women to strengthen timely pregnancy awareness include the provision of free pregnancy test kits for home use, routine pregnancy testing by visiting community health workers and outreach education programs [8–10].

In the last decade there have been few accounts from women in middle-income countries describing their lived experiences from first suspicion of pregnancy to confirmation [11–13].

Healthcare providers’ perspectives on reasons for delay in care-seeking have been described in the literature, but there has been little exploration into their opinions on ways to strengthen early recognition of pregnancy [14, 15]. In this study, we aim to explore experiences of pregnancy recognition and confirmation among South African women obtaining abortion care after 9 weeks gestational age, as well as abortion providers’ and policy makers’ suggestions for strategies to facilitate early pregnancy confirmation.

## Methods

### Study setting

We conducted the study in two health subdistricts in the Western Cape Province, South Africa: The City of Cape Town metropole (urban) and the Eden district (rural) approximately 500 km away. The principal investigator (female) and trained research assistant (female) recruited participants from July through September 2017, at an urban non-governmental organization (NGO) providing surgical abortion care in the first and second trimester and at two public sector hospitals (urban and rural) providing second trimester surgical abortion care. Both interviewers are experienced researchers in sexual and reproductive health and rights and women’s rights to access a safe abortion within the South African context. In combination, the selected sites reflect the types of services in the Western Cape providing abortion care, all have a large case volume and provide care to a representative section of the population that use public sector or NGO abortion services. Abortion services are free of charge at these facilities for women referred from public primary healthcare facilities. We excluded facilities that provided solely medical abortion up to 9 gestational weeks as presentation for care could not be considered delayed, and hospitals providing second trimester medical abortion due to low volume.

### Study participants

Women eligible for abortion and awaiting their procedure were approached by the research assistant in the abortion facility reception areas and invited to participate in the study. Forty respondents were purposively selected to include younger women (< 36 years), for the most part having their abortion at 13–20 weeks gestational age (GA) (n = 34); the remainder were 9–12 weeks GA (n = 6). We targeted younger women as they are generally over-represented among women experiencing delays in accessing abortion care [16]. Similar numbers were recruited from the 3 sites: rural (n = 10) and urban (n = 16) public services, and the urban NGO (n = 14). All respondents at public sector facilities were 13–20 weeks GA, those attending NGOs were between 9 and 20 weeks GA.

Staff participants (n = 7) included 3 registered nurses or midwives, 1 physician and 1 consultant providing second trimester surgical abortion, all provided services at the facilities where women were recruited. In addition, we interviewed 2 policy makers in provincial structures responsible for sexual and reproductive health services.

### Study design and data collection

We used a qualitative research design informed by a thematic analysis [17]. The study principal investigator (DC) and the research assistant conducted written informed consent, in-depth interviews and group discussions with women in a private space in the abortion facility following recovery after the abortion procedure and prior to discharge from the facility. We performed 15 individual in-depth interviews and 8 group discussions among 25 women with group size varying between three and four women. Most women preferred in-depth interviews (IDIs) over group discussions (GDs) to avoid waiting for a group to assemble and to safeguard their privacy. DC interviewed staff individually at their place of work or by phone, according to their preference.

All women completed a short questionnaire on pregnancy recognition with assistance from the research assistant if needed. For IDIs and GDs, we used a semi structured interview guide with open-ended questions and probing. Our topic guide was informed by earlier work on pregnancy awareness and key research objectives [7, 18]. Key issues explored included: The experience of pregnancy discovery and perception of risk of pregnancy; familiarity with, and barriers to obtaining and doing a pregnancy test; knowledge of fertile periods within the menstrual cycle and when to first test for pregnancy after unprotected sex. Questions for staff centred on their opinion about public health interventions that might assist women to recognize and confirm an unintended pregnancy promptly.

We piloted interview guides with role players to achieve good understanding and flow of questions and further modified questions as new issues emerged during data collection. We conducted interviews in English or in Afrikaans according to participant’s preference. In-depth interviews and group discussions lasted between 20 and 40 min, were tape-recorded and transcribed verbatim, and then translated into English. Confidentiality and anonymity were ensured in IDIs, although there was sharing of opinion in the GDs.

### Ethical considerations

Given the sensitivity of the subject matter interviewers took care to create an empathetic and supportive experience for women. Women participants were reimbursed ZAR100 to compensate for their time, staff were not compensated. All data were stored in locked files and password protected computer files. Recordings were deleted once they had been checked after data transcription. The study protocol was approved by the University of Cape Town Health Sciences Human Research Ethics Committee (HREC Ref: 245/2017), the Western Cape Provincial Department of Health (WC2017RP40_636) and the NGO clinics.

### Data analysis

We analysed the data using a thematic analysis approach in which main themes and categories were identified and analysed within and across data. The process of thematic analysis involved the following steps: (i) Familiarisation with the data, (ii) preliminary coding, (iii)) search for patterns or themes across the different interviews, (iv) review, defining and naming themes, and (v) reporting of results [17]. We used Nvivo version 12 [19] to facilitate the sorting and data management process. Initial categories for coding were drawn from the interview guides by investigator OS. To check against coder variation, transcripts were randomly selected and crosschecked for author’s coding accuracy, any discrepancies were resolved by discussion between investigators (OS, DC) and final codes were assigned.

## Results

### Women participants

Data saturation was achieved after interviewing 40 study participants with no new information to emerge. The mean age of the 40 women participants was 25 years (SD: 4.5 years). The majority (28) had competed their high school education or beyond, but 23 had no paid work and 7 depended on a social grant for income. Most (31) had one or more prior pregnancies, five had prior abortions. Half (21) did a pregnancy test at home; the remainder had the first test for this pregnancy at a health facility. Half (20) had partners who were supportive of their decision to abort this pregnancy. Gestational age at time of pregnancy confirmation was more than 7 weeks for majority (26). Half (22) had used the injectable, 13 had used dual contraceptive methods (injectable and condoms) in the past and 6 had no prior usage of any method.

Three main themes with subthemes emerged in the interviews and discussions with women participants. Main themes included pregnancy recognition, experience with confirming the pregnancy and women’s knowledge of fertility, the menstrual cycle and when to test for pregnancy.

### Pregnancy recognition

#### Women’s own recognition of pregnancy

Although many women detected pregnancy signs and symptoms, often they did not link them immediately with the possibility of being pregnant and waited to gain better clarity on how to interpret them. Postponement was often a consequence of having previously missed a period without being pregnant, having irregular periods or not keeping track of their menstrual cycle.

*Well, I definitely thought that maybe my, my period has skipped a month because why,* *normally it does that sometimes, it skips a month or 2 and then it comes back again…but then when the second month came and I still didn’t get.. my period, that’s when I started realising okay, it could be possibility that I might be pregnant.” (IDI)*

#### The role of social networks or family and friends in recognizing this pregnancy

Women mentioned that others, including family members, friends, partners or work colleagues noticed pregnancy signs and symptoms. This recognition of pregnancy by others in their social networks frequently acted as a catalyst for women to seek confirmation of pregnancy. Some recounted very supportive actions by their partner in helping them do their first test.

*“…yes, my boyfriend did suggest to me [to take a pregnancy test] and he told me that yoh I sleep a lot…I would have not done that [tested, had he not encouraged me] …because I was scared [of the pregnancy] …I needed someone to talk to me and then he did talk to me……he was the one who was the one doing the pregnancy test.” (GD)*

Others said that family or friends noticed their pregnancy symptoms and prompted them to take a pregnancy test.

*“My mommy, coz she tells me….* [name], *you must go get checked out coz you skipped a period… and you very moody sometimes so maybe you pregnant, you must go find out” (IDI)*

*“My friends said to me, ‘you are pregnant’ because I had moods.” (IDI)*

#### The prospect of pregnancy, hesitation to confirm pregnancy

Women elaborated on their emotional conflict with respect to the pregnancy, their worries, anxieties and indecision around what to do and how a pregnancy would affect their life choices. Reasons for initial hesitation in seeking confirmation by testing varied with some preferring to wait for their period to commence while others were worried about how partners might react. These experiences link into the disclosure/nondisclosure component of Coast’s framework [6]. Initial delays ranged from one week to three months.

*“I was very angry [when I suspected I was pregnant] coz I, I did prevent it even though I knew that I missed, I skipped my pills but then there was a time that I thought like I want, I want, maybe I’m going to keep the baby because it’s a blessing. But then on the other side I thought of myself and I thought of my life and I’m not ready [Participant reported waiting 2 months before testing] (IDI)*

*“Yes I was, I was a bit scared [to test] because I already have 2 kids so I didn’t know how my mom and them would take it and things like that, so I was going through emotional time...” [Participant reported waiting 3 months before testing] (IDI)*

### Experiences with confirming the pregnancy

#### Home- versus clinic-based testing

Just over half of the participants tested at home. Many felt compelled to self-test for reasons including taking responsibility for their own bodies or being concerned about the passage of time and missed periods. In contrast, reasons for preferring to test at a health care facility (HCF) included being afraid to confirm pregnancy alone; concerns about doing tests incorrectly and considering it a waste of time and money knowing they would ultimately need to confirm at the clinic.

*“I felt like it, it’s my own responsibility to, to check, to know what is happening on my body. Especially since I noticed that I missed my period because I was waiting for my period and then when I didn’t come I started to, to panic and, and I thought I should go with, without consulting anyone.” (GD)*

*“I’m scared to find out myself, I don’t know how to react, it’s better when there is someone, a professional who knows these, these things.”* *(GD)*

#### Cost and quality of tests

Women commented on the variation in cost of home pregnancy tests and raised concerns about the accuracy of the more affordable tests. Many purchased multiple (two to three) home pregnancy tests to confirm their pregnancy before visiting a health care facility. Reasons for multiple tests included wanting to be certain about and not trusting the result(s). Some reported disbelief at the result and wanted to seek definitive confirmation at a health care facility.

*“I did it at home…I had to be like certain, certain, certain so I had to buy the most expensive test that I could get. And then, …because I was in disbelief the first time I got a more expensive one and it was so much money because I didn’t believe that I was pregnant. But then to make sure I made sure that I bought the most expensive ones which would equal it’s more trustworthy… I went to a clinic immediately the day after because I still didn’t trust the pharmacy test… I was shocked…the first thought was I’m not the problem, the test is the problem.”* *(GD)*

### Knowledge of fertility, the menstrual cycle and when to test for pregnancy

#### Timing of the fertile period

Some women demonstrated accurate knowledge about fertility and menstrual cycle events. They seemed familiar with the word “ovulating” and understood that this meant they were more likely to fall pregnant. Some knew about the “fertile window” but admitted not keeping track of their cycle, but for the most part there was limited understanding about fertility of risk of pregnancy.

*“…on day 14 of your cycle your egg gets released from your ovaries and then, it’s, that’s the most fertile time coz then the sperm can go and fertilise it because it’s been released into the fallopian tubes so ja, that’s it, day 14, if you count your cycle you should know but who does, I don’t…”* *(GD)*

*“I don’t know anything about that* [fertility period] *but the only thing that I think or, is that when you are in your periods you are more likely to get pregnant.”* *(IDI)*

“*I thought it* [fertility period] *was a little before or after your period.”* *(IDI)*

#### When to test for pregnancy

Most participants confided not knowing about optimal timing for early pregnancy testing. Some thought testing should wait until physical changes became apparent. Many thought testing could be done as soon as five days after having unprotected sex whilst others thought it better to wait one week to a month.

*“I know it’s a couple of days or a week* [after unprotected sex you can test for pregnancy], *I think, I’m not sure anymore.”* *(IDI)*

*“I thought it was too soon take the test after I’d missed my first period because I thought maybe it was coz of the sickness and, and coz I’d missed 1 before because of that and so I thought to give it a bit more time because I knew if you do test too early it’s kind of irrelevant.”* *(IDI)*

### Providers’ and policy makers opinions

#### Strengthening reproductive awareness

Health care providers and policy makers suggested a range of interventions aimed at strengthening reproductive awareness generally and specifically self-awareness around the likelihood and prompt recognition of pregnancy. Abortion providers acknowledged the efforts by government with a national campaign aimed at empowering young women and adolescent girls but stressed that more is needed to reach a broader audience through schools, television and other media channels.

*“I’ve always said there’s not a link between missing your period and think ah, maybe I’m pregnant, it isn’t there….there should be much better sexual education at schools I think the, there should be the, the TV, the radio the, the papers, magazines, it should be all over.” (Physician, abortion provider to public and NGO sectors)*

*“Firstly there’s a need to increase awareness of the risk of being pregnant particularity if a woman is having unprotected intercourse – that would be self-awareness… then there’s a misunderstanding in the community that you need to see a doctor to confirm the pregnancy … if everybody just knew that ovulation comes before menstruation and you don't have to give the contraceptive time to work out of your system…. that in actual fact will prevent a lot of pregnancies or you'll diagnose them sooner…, so, so I think schools and I think just common misunderstandings.”* *(Policy maker, provincial government)*

*“…women need to know about their menstrual cycle, they need to know about return to fertility off their contraceptive, how does a contraceptive work.”* *(OBGYN consultant abortion provider, public sector)*

#### Removing barriers to in-clinic testing

Some providers highlighted the need for easier access to pregnancy tests within clinics. A suggestion was to have readily accessible free pregnancy tests in dispensers located in clinic spaces as a mechanism to overcome clinic-based barriers such as the need to open a folder and lack of privacy.

*“well nobody waits in a, queue if they come in for condoms because you’ll find condom boxes in the entrance of the facility, you’ll find condom boxes in the toilets, you’ll find condom boxes in the counters where the clerks are and people walk in. But when it comes to a pregnancy test unfortunately, you have to take a folder out for you.” (Nurse abortion provider, public sector)*

*“Firstly there’s a need to increase awareness of the risk of being pregnant particularity if a woman is having unprotected intercourse – that would be self-awareness…. Secondly would be access… there’s a misunderstanding in the community that you need to see a doctor to confirm the pregnancy. That is incorrect… a commercially available urine pregnancy is relatively accessible and cheap…its an access point.” (Policy maker, provincial government)*

*“I would imagine that it should be something that any woman could walk in and say I just want, go to, you know to like the triage room or the family planning room and say I’d like a pregnancy [test] and she could take it and test without anyone knowing. Coz that would also be, so I think ease, not having to open a folder, not having to explain yourself to somebody and then also not having to divulge the test result to anybody. So that would be useful rather than somebody else having to test it for you, because I think especially for people in the community who might know people they don’t always want everybody to know that they’re pregnant.”* *(OBGYN consultant abortion provider, public sector)*

#### Expanding access to free testing and counselling beyond the clinic

Other ideas were to utilize community-based services to raise awareness around early pregnancy testing, to expand access to testing at community level, and provide health information through these channels, when appropriate. In addition, it was suggested to institute free pregnancy testing in local pharmacies that employ an on-site nurse. This could easily be implemented in the pharmacy chains with existing private–public partnerships, and costs could be expected to be the same as the current in-clinic testing service.

*“the NGOs and community health workers, home based carers … We should train them to do a pregnancy test or to show people how to do their own pregnancy test …then she would get the necessary information, she would get the, the necessary referral, where to go to. Also added in, added health education on if she’s pregnant.”* *(Policy maker)*

*“Another option would be to get free state pregnancy tests at private pharmacies because there are, pregnancy tests at pharmacies are actually very expensive for the average person on the street so, for example like [names of pharmacies with existing public -private partnerships], … they often have a nurse there with government stock of family planning, would they have government stock of pregnancy tests there… it should be feasible because the tests are there already so I mean I can’t imagine that the cost would go up exponentially because pretty much every woman will eventually present to some place.”* *(OBGYN consultant abortion provider, public sector)*

## Discussion

Women’s descriptions of their emotional conflict prior to pregnancy confirmation are coherent with Coast’s framework for trajectories to abortion care [6]. Many adopted a “wait and see” approach and denial of pregnancy and expressions of fear and anxiety were also prevalent, although all women in our study were able to obtain a safe and legal abortion.

Our findings highlight the importance of women’s social networks in pregnancy recognition. Women described how family or friends played a key role in recognizing and then prompting them to confirm the pregnancy, similar to reports in a much earlier US-based study [7]. While not a finding in this study, it is worth noting that the benefit of others’ support in encouraging women to test for pregnancy could be negated if that role extended to coercion around women’s decision-making regarding the pregnancy, as has been described elsewhere [20].

Although women’s reactions to an unplanned pregnancy have been described as individual and not predictable [21], internal assets such as knowledge around accurate timing for pregnancy testing or supportive social networks can be expected to assist with timely care-seeking, or avoidance of delay. For women preferring to see a health care provider to confirm their pregnancy status, the onerous process at the clinic as well as negative healthcare provider attitudes are barriers in this setting and have also been reported elsewhere [22].

Abortion care providers and other stakeholders in sexual and reproductive health (SRH) programs agree that the absence of accessible and affordable or free pregnancy tests significantly affects women’s lives, and that without ready access to these tests, women are less likely to confirm a pregnancy promptly. Providing pregnancy tests through community-based programs has been cost-effective in low-income settings [23] and can be combined with information dissemination around SRH.

Self-care interventions provide a mechanism to relieve the growing burden on healthcare services as experienced globally [24]. Providing free and readily available pregnancy tests for women to self-test as proposed by providers and policy makers in this study aligns well with the WHO self-care conceptual framework and could additionally strengthen women’s autonomy and agency [24]. Recent research on the feasibility and acceptability of routine self-testing for early recognition of pregnancy has been piloted in our setting [25], however more studies are needed to evaluate the effectiveness and costs of such approaches.

As a strength, our study included women recruited from a wide range of services, both urban and rural as well as public and NGO services. However a limitation is that we included only women who gained access to services and not those who had been denied abortion care, as these women constitute a very hard-to-reach population.

## Conclusion

Early recognition of unintended pregnancy in this setting is frustrated by poor understanding and awareness of fertility and pregnancy signs and symptoms, compounded by a distrust of commercially available tests. Promoting greater public awareness of early pregnancy signs and symptoms and of the importance of prompt testing are needed to achieve early pregnancy recognition. Having free pregnancy tests more readily available in both the clinic and the community environment may help women with unintended pregnancy confirm their pregnancy status promptly.

## Data Availability

De-identified data will be made available on reasonable request.

## Abbreviations

NGO: Non-governmental organization
SRH: Sexual and reproductive health
GA: Gestational age
IDI: In-depth interview
GD: Group discussion
ZAR: South African rands
SD: Standard deviation
HCF: Healthcare facility
WHO: World Health Organisation
